# Integrative effects of transcutaneous auricular vagus nerve stimulation on esophageal motility and pharyngeal symptoms via vagal mechanisms in patients with laryngopharyngeal reflux disease

**DOI:** 10.3389/fnins.2024.1287809

**Published:** 2024-03-07

**Authors:** Yizhou Huang, Jie Liu, Chaolan Lv, Chenyu Sun, Muzi Meng, Scott Lowe, Yue Yu

**Affiliations:** ^1^Department of Gastroenterology, The PLA Navy Anqing Hospital, Anqing, Anhui, China; ^2^Department of Gastroenterology, Division of Life Sciences and Medicine, The First Affiliated Hospital of USTC, University of Science and Technology of China, Hefei, Anhui, China; ^3^Department of General Surgery, The Second Affiliated Hospital of Anhui Medical University, Hefei, Anhui, China; ^4^Bronxcare Health System, New York, NY, United States; ^5^College of Osteopathic Medicine, Kansas City University, Kansas City, MO, United States

**Keywords:** vagus nerve stimulation, neuromodulation, autonomic function, heart rate variability, laryngopharyngeal reflux disease

## Abstract

**Background and aim:**

Laryngopharyngeal reflux disease (LPRD) is primarily characterized by discomfort in the pharynx and has limited treatment options. This research aimed to assess the efficacy of transcutaneous auricular vagus nerve stimulation (tVNS) in patients with LPRD and delve into the potential underlying mechanisms.

**Methods:**

A total of 44 participants, diagnosed with LPRD were divided into two groups randomly. Twice-daily stimulation was delivered for 2 weeks for patients in experimental group, with stimulation ranging from 1.0 mA to 1.5 mA (*n* = 22), while the control group underwent sham tVNS (*n* = 22) with the same stimulation parameters and different anatomical location. The severity of symptoms and levels of anxiety and depression were monitored using questionnaires. High-resolution esophageal manometry data were collected, and the patients’ autonomic function was assessed through heart rate variability analysis.

**Results:**

There was a positive correlation between reflux symptom index (RSI) scores and low frequency/high frequency (LF/HF) ratio (r = 0.619; *p* < 0.001), Hamilton anxiety scale (HAMA) scores (r = 0.623; *p* < 0.001), and Hamilton depression scale (HAMD) scores (r = 0.593; *p* < 0.001). Compared to the pre-tVNS phase, RSI (*p* < 0.001), HAMA (*p* < 0.001), and HAMD (*p* < 0.001) scores were significantly reduced after 2 weeks of treatment. Additionally, the resting pressure of the upper esophageal sphincter (UESP; *p* < 0.05) and lower esophageal sphincter (LESP; *p* < 0.05) showed significant enhancement. Notably, tVNS led to an increase in root mean square of successive differences (RMSSD; *p* < 0.05) and high frequency (HF; *p* < 0.05) within heart rate variability compared to the pre-treatment baseline. Compared to the control group, RSI (*p* < 0.001), HAMA (*p* < 0.001), and HAMD (*p* < 0.001) scores in tVNS group were significantly lower at the end of treatment. Similarly, the resting pressure of UESP (*p* < 0.05) and LESP (*p* < 0.05) in tVNS group were significantly higher than that of control group. Notably, RMSSD (*p* < 0.05) and HF (*p* < 0.05) in tVNS group were significantly higher than that of control group.

**Conclusion:**

This study demonstrated that tVNS as a therapeutic approach is effective in alleviating LPRD symptoms. Furthermore, it suggests that improvements in esophageal motility could be associated with vagus nerve-dependent mechanisms.

## 1 Introduction

Laryngopharyngeal reflux disease (LPRD) is characterized by the regurgitation of stomach contents into the larynx and pharynx, leading to an inflammatory response in the mucous membranes of the throat. This inflammatory process manifests as a range of symptoms, including hoarseness, persistent cough, difficulty swallowing (dysphagia), and the sensation of a lump (globus) in the throat (Mishra et al., 2020). The condition significantly diminishes the quality of life for affected individuals and increases their susceptibility to various laryngeal disorders, such as reflux laryngitis, subglottic stenosis, laryngeal cancer, granulomas, contact ulcers, and vocal cord nodules (Falk and Vivian, 2016). It is recognized that 24-h pH-metry is the gold standard test for diagnosing LPR (Mishra et al., 2020). But it is an expensive and time consuming investigation. Thus there are some simplified approaches for monitoring and quantifying the symptoms, such as Reflux Symptom Index (RSI), Reflux Finding Score, CarlssonDent, ReQuest, GerdQ, etc. (Zhang et al., 2023).

The upper esophageal sphincter (UES), a crucial barrier against reflux, maintains a close relationship with the vagus nerve, while disturbances in autonomic function have been associated with the development of LPRD (Benjamin et al., 2017; Wang et al., 2019). Although proton pump inhibitors (PPIs) have gained recognition as a therapeutic approach for LPRD, only around half of patients respond to PPI treatment, and some experience minimal relief in symptoms when compared to a placebo (Steward et al., 2004). Therefore, there is a need for exploring complementary and alternative medicine (CAM) as a treatment for LPRD. A recent study found that transcutaneous electrical acupoint stimulation (TEAS) combined with PPI showed a significantly greater improvement in LPRD symptoms (Shen et al., 2023).

Transcutaneous auricular vagus nerve stimulation (tVNS), an alternative to invasive vagus nerve stimulation (IVNS), has found utility in addressing functional gastrointestinal disorders (FGID), inflammatory bowel disease (IBS), and other conditions (Ventureyra, 2000; Carreno and Frazer, 2016; Keute et al., 2018; Hong et al., 2019). The auricular branch of the vagus nerve extends to the cymba concha in the outer ear. By non-invasively stimulating this region, inflammation can be suppressed, and vagal activity enhanced (Steward et al., 2004). The present study endeavors to assess the impact of tVNS on alleviating LPRD symptoms and improving esophageal function. Furthermore, the investigation seeks to elucidate the potential autonomic mechanisms underlying these effects in individuals afflicted by LPRD.

## 2 Patients and methods

### 2.1 Study participants

This preliminary investigation employed a randomized, single-blind, and sham-controlled approach to assess the effects of transcutaneous auricular vagus nerve stimulation (tVNS) in individuals diagnosed with laryngopharyngeal reflux disease (LPRD). Notably, the participants did not participate directly in shaping the study’s design, recruitment, or implementation. Inclusion criteria consisted of individuals aged 18 to 65 years, possessing a reflux symptom index (RSI) score exceeding >13, having been diagnosed with LPRD for a duration surpassing 3 months, and demonstrating a willingness to adhere to the stipulated treatment regimen.

Exclusion criteria encompassed conditions such as diabetes, malignancies, respiratory disorders, endocrine abnormalities, cardiac ailments, upper gastrointestinal afflictions, or other significant systemic maladies. Additionally, those who had recently used medications with the potential to impact autonomic function or acid suppression, individuals engaged in prolonged smoking or excessive alcohol consumption, women experiencing menstruation, pregnancy, or lactation, and subjects exhibiting allergies to the electrodes were excluded from participation. Within the confines of these criteria, a total of 44 LPRD patients were recruited to partake in the investigation, each providing informed consent prior to their involvement.

Ethical considerations were rigorously upheld, and the study was granted approval by the Ethics Committee of The First Affiliated Hospital of USTC (2016 L36).

### 2.2 Study protocol

Forty-four participants were allocated into two groups through a randomized allocation, maintaining an equitable 1:1 ratio. The random assignment was executed using a random digital table within the Statistical Package for the Social Sciences (SPSS) software. Prior to undergoing their designated interventions, individuals within each group were instructed to complete a battery of three distinct questionnaires, namely the 14-item Hamilton Anxiety Scale (HAMA), the 17-item Hamilton Depression Scale (HAMD), and the Reflux Symptom Index (RSI). Furthermore, comprehensive evaluations were conducted, encompassing a high-resolution esophageal manometry (HREM) examination and continuous electrocardiogram (ECG) monitoring.

**Figure 1:**
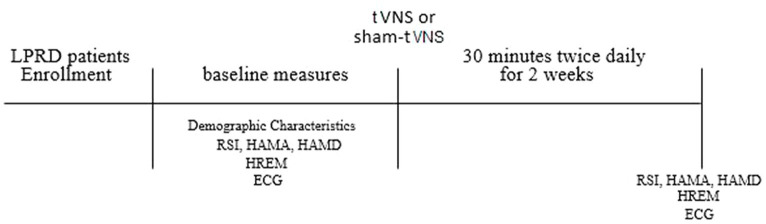
Study protocol. LPRD, Laryngopharyngeal reflux disease; tVNS, transcutaneous auricular vagus nerve stimulation; RSI, reflux symptom index; HAMA, 14-item Hamilton Anxiety Scale; HAMD, 17-item Hamilton Depression Scale; HREM, high-resolution esophageal manometry; ECG, electrocardiogram.

Following the initial assessments, participants were subjected to either tVNS or sham tVNS sessions, with a frequency of twice daily for a duration of 2 weeks within clinic. To ensure impartiality and mitigate potential subjective biases, the post-treatment evaluations of all subjects were carried out by an investigator who had no prior involvement during the stimulation phase. The content of the follow-up assessment remained consistent with the baseline evaluation, while meticulous attention was directed toward assessing the safety and tolerability of the interventions. The procedural outline of this study is elucidated in Figure 1.

### 2.3 tVNS and sham-tVNS treatment

**Figure 2:**
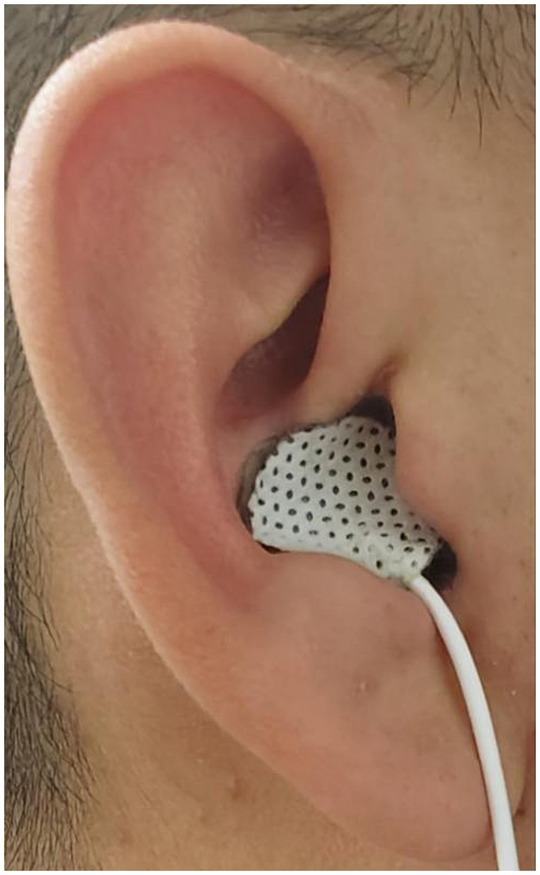
Overview of stimulation methodology and location.

The bilateral auricular concha regions exhibit a rich distribution of the vagus nerve. Prior research has documented that stimulating the concha regions of both ears can yield enhancements in esophageal motility and elevation of vagal activity. Consequently, in this study, tVNS was conducted on the bilateral auricular concha areas, a method supported by previous findings (Zhu et al., 2021; Long et al., 2022; see Figure 2). The stimulation point for tVNS was at cavity concha, while sham point was at the earlobe. The cavity concha was found to be solely innervate by the vagus nerve and great auricular nerve in earlier studies (Peuker and Filler, 2002).

Preceding the stimulation procedure, the auricular skin was meticulously disinfected using alcohol. Subsequently, a pair of surface electrode pads were precisely positioned on the bilateral concha areas. Stimulation was administered employing a watch-sized stimulator (SNM-FDCM01, Ningbo Maida Medical Device, Inc., Ningbo, China). In parallel, the sham tVNS intervention was conducted at a distinct anatomical location (Zhang et al., 2018). tVNS sessions lasting 30 min. Noteworthy is the uniformity of stimulus parameters across both interventions: pulse trains alternating between 2 s of stimulation and 3 s of rest, a pulse width measuring 0.5 ms, a pulse frequency set at 25 Hz, and a pulse amplitude ranging from 1.0 mA to 1.5 mA, determined based on patient tolerability and preference. To eliminate any potential bias, all participants remained unaware of the specific treatment modality being administered (Zhu et al., 2021). A stimulation of 2/3 s ON/OFF cycle was selected owing to its effectiveness in enhancing GI motility in a previous study (Shi et al., 2021).

### 2.4 Measurements

#### 2.4.1 Assessment of RSI, HAMA, and HAMD

The RSI was employed as both a diagnostic and evaluative tool for patients with LPRD (Belafsky et al., 2002). Succinctly, RSI gauges the severity of symptoms experienced by individuals over the preceding month, utilizing a scoring system that assigns values ranging from 0 (indicating no issue) to 5 (representing a severe problem) for each of its nine constituent items. Higher cumulative scores correspond to more pronounced symptomatology, with the maximum potential overall score reaching 45. In this study, RSI was modified to only account for the previous 2 weeks.

Meanwhile, the HAMA and HAMD have established themselves as widely utilized instruments for assessing the manifestation of anxiety and depression within clinical contexts (Hamilton, 1959; Hamilton, 1960). HAMA encompasses 14 distinct items, each rated on a scale spanning from 0 (absent) to 4 (severe), culminating in a cumulative score range of 0 to 56. In contrast, the HAMD comprises 17 items, contributing to a maximum total score of 62. These aforementioned questionnaires have consistently demonstrated commendable reliability and validity in effectively appraising psychological states (Pappa et al., 2020; Yang et al., 2022).

#### 2.4.2 High-resolution esophageal manometry

Esophageal motility analysis was conducted using a water-perfused esophageal manometric catheter furnished with 24 pressure sensors distributed at 1 cm intervals (MedKinetic, Ningbo, China). The procedural protocol encompassed a sequence of key steps, commencing with two initial baseline recordings conducted in the absence of swallowing. Subsequent to this, the examination progressed with the administration of 10 swallows of 5 mL of water, followed by the execution of two consecutive swallows involving 2 mL of water, each completed within a span of 5 s. For the analysis of HREM parameters, a dedicated suite of analytical software (Medview 360) was deployed for comprehensive assessment (Yu et al., 2019).

During the process of swallowing, discrete aspects of esophageal manometry data were gathered from each patient, incorporating variables such as upper esophageal sphincter pressure (UESP), upper esophageal sphincter length (UESL), lower esophageal sphincter pressure (LESP), lower esophageal sphincter length (LESL), contraction front velocity (CFV), distal latency (DL), distal contraction integral (DCI), and integrated relaxation pressure (IRP). The median DCI is recognized as a marker of esophageal contractile vigor. IRP represents mean esophagogastric junction (EGJ) pressure measured with an electronic equivalent of a sleeve sensor for four continuous or non-continuous seconds of relaxation in the 10-s window following deglutitive UES relaxation. DL is the interval between UES relaxation and the contractile deceleration point. CFV refers to the slope of the tangent approximating the 30 mmHg isobaric contour between the proximal pressure trough and the CDP.

#### 2.4.3 Assessment of autonomic functions

The ECG recordings (CT-082, Hangzhou Baihui Electrocardiograms, China) with 12 leads and 10 electrodes yielded short-term (5-min) heart rate variability (HRV) data, which underwent thorough processing using the HRV analysis software (Cardiotrak Holtersystem version: 1.2.0.0, Hangzhou Baihui Electrocardiograms, China). Our attention is directed toward two pivotal time-domain HRV measurements: the Standard Deviation of the normal-to-normal intervals (SDNN) and the Root Mean Square of Successive Differences (RMSSD). In addition, an exploration of frequency-domain HRV parameters was undertaken, encompassing total power (TP) in the frequency range of 0.00 to 0.40 Hz, low-frequency (LF) power spanning 0.04 to 0.15 Hz, high-frequency (HF) power within the 0.15 to 0.40 Hz spectrum, the ratio of high-frequency power to total power (HF% or HF/Tot), and the ratio of low-to-high frequency power (LF/HF).

Of note, RMSSD, HF, and HF% primarily serve as indices of vagal activity, while SDNN and TP predominantly reflect the composite autonomic nervous system activity involving both sympathetic and vagal components. The LF parameter is modulated by both sympathetic and vagal influences, with sympathetic predominance. On the other hand, LF/HF offers insights into the equilibrium between sympathetic and vagal innervation (Baldissarelli et al., 2017; Wang et al., 2018).

### 2.5 Statistical analyses

For all statistical analyses, SPSS version 22.0 software was employed. Descriptive statistics for quantitative variables were presented as mean values accompanied by their corresponding standard deviations (SD). Changes in the data pre- and post-treatment were evaluated utilizing a paired t-test, while the difference between the two groups were tested by using independent t-test. Normality test was conducted using Shapiro–Wilk test. The t-tests and Pearson correlation tested were appropriate for analyzing data. Categorical variables were expressed as rates and subjected to analysis using the χ-2 test. The threshold for statistical significance was set at a *p*-value less than 0.05. Furthermore, to explore relationships between variables, correlation analysis was executed employing Pearson’s correlation test.

## 3 Results

### 3.1 Patient characteristics

The stimulation procedure did not give rise to any adverse events, including but not limited to occurrences such as headache, lightheadedness, tinnitus, tachycardia, or rash. A total of 45 LPRD patients were recruited, while a single participant dropped out due to contracting an upper respiratory virus infection during the course of treatment. This individual exhibited improvement subsequent to receiving antiviral medication. The comprehensive demographic and clinical data of the 44 subjects who successfully completed the course of tVNS or sham-tVNS intervention are meticulously presented in Table 1. Examination of variables encompassing sex, age, body mass index (BMI), and duration of the disease revealed notable similarity between the tVNS and sham-tVNS groups.

**Table 1:** Baseline characteristics of patients treated with tVNS or sham-tVNS.

	tVNS (*n* = 22)	sham-tVNS (*n* = 22)	t/x^2^	*p*-value
Male, n (%)	10 (45.45%)	10 (45.45%)		1
Age (yr)	45.50 ± 10.84	48.27 ± 10.02	−0.881	0.383
BMI (kg/m^2^)	22.19 ± 1.46	21.71 ± 1.00	1.263	0.214
Duration (months)	20.82 ± 3.71	19.19 ± 4.32	1.344	0.186

tVNS, transcutaneous auricular vagus nerve stimulation; BMI, body mass index.

### 3.2 Effects of tVNS and sham-tVNS on RSI, anxiety and depression

**Figure 3:**
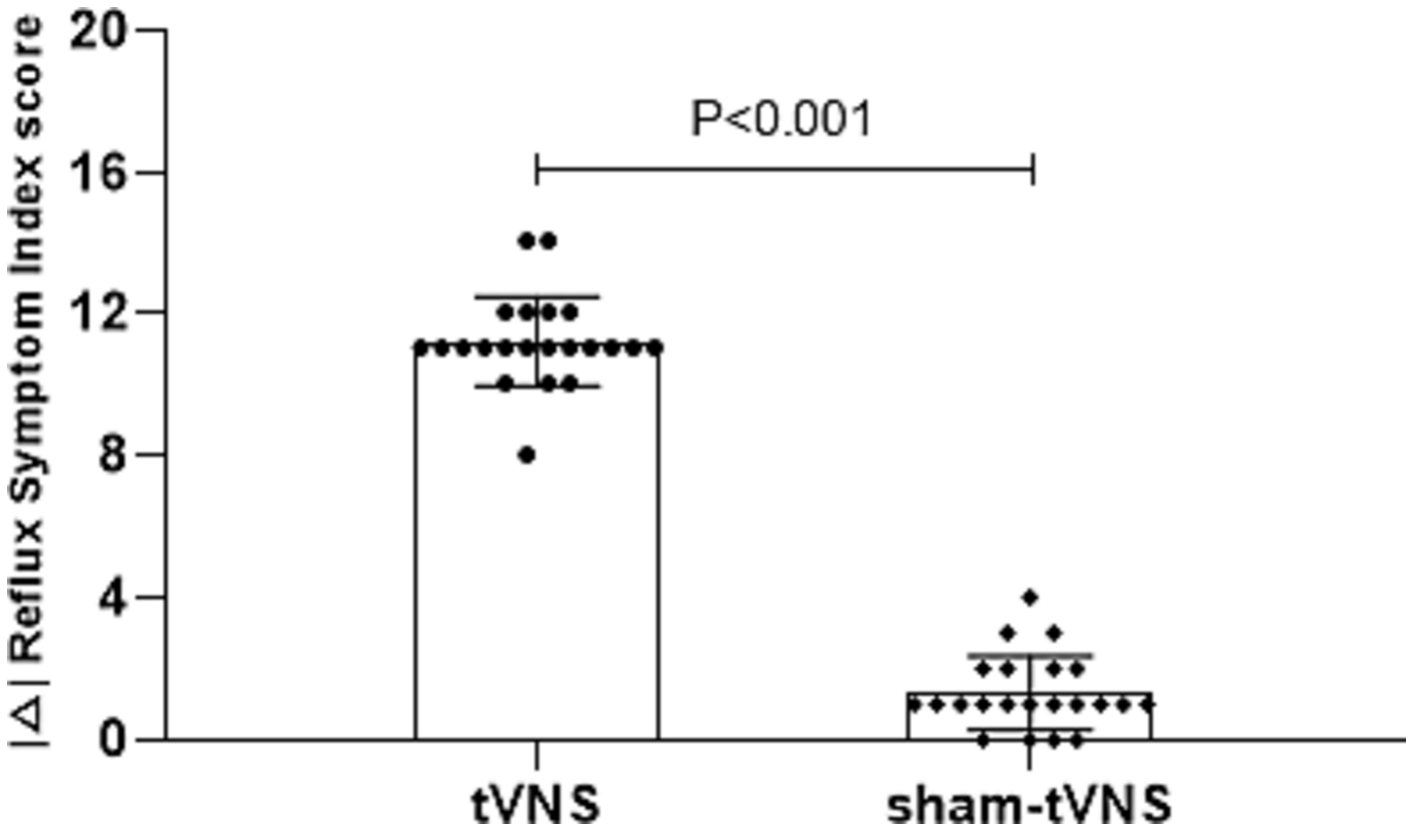
The effect of tVNS (*n* = 22) compared with sham-tVNS (*n* = 22) on delta RSI score. ^*^p < 0.05 vs. before treatment.

**Figure 4:**
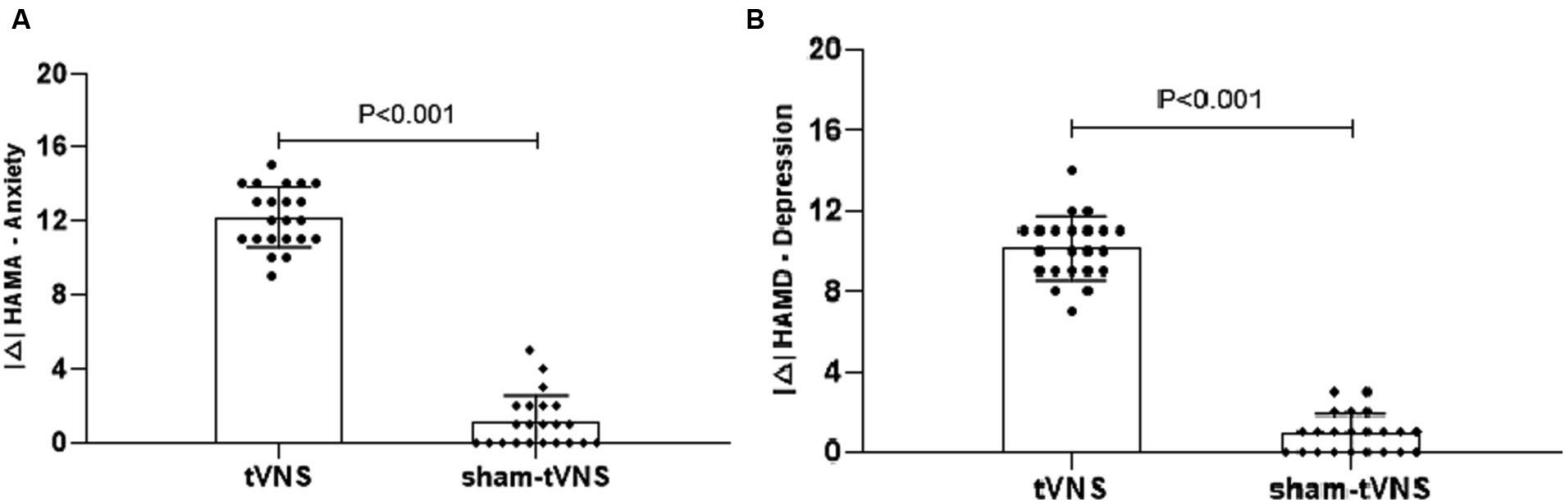
**(A)** The effect of tVNS (*n* = 22) compared with sham-tVNS (*n* = 22) on delta HAMA score. ^*^p < 0.05 vs. before treatment. **(B)** Effect of tVNS (*n* = 22) and sham-tVNS (*n* = 22) on delta HAMD score. ^*^p < 0.05 vs. before treatment.

**Figure 5:**
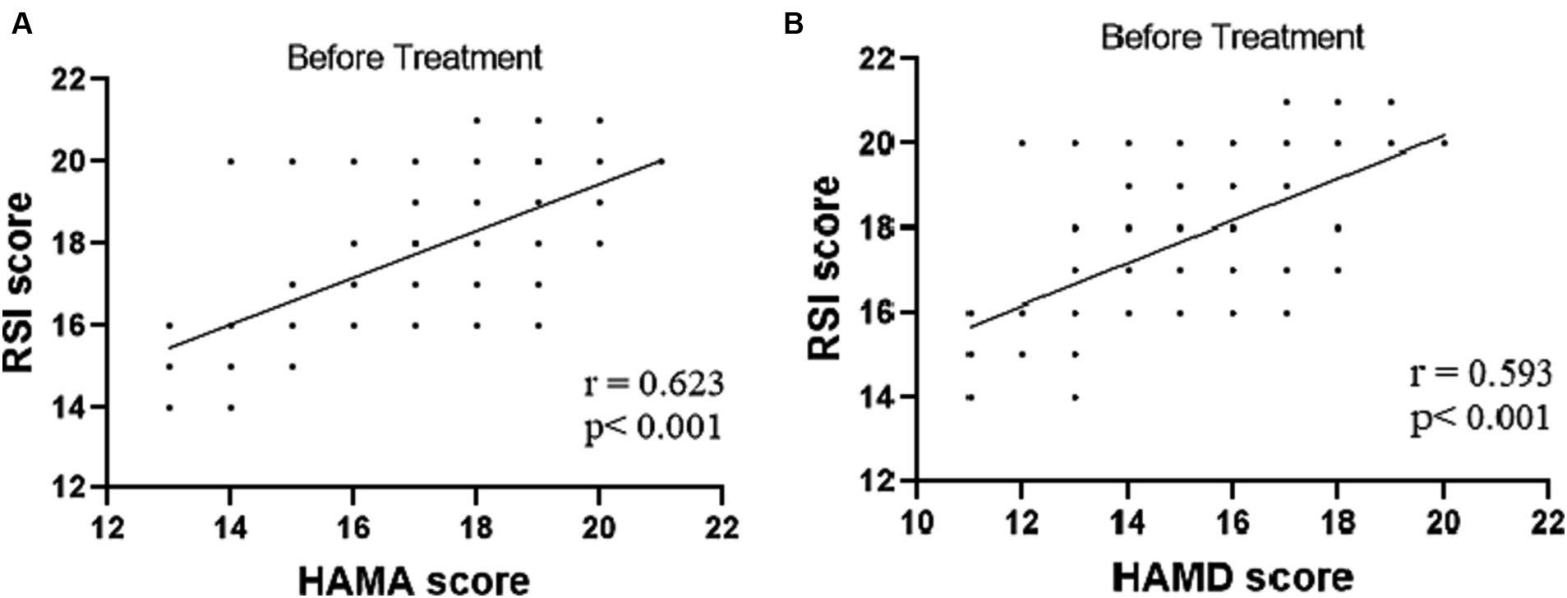
**(A)** Correlation of HAMA score with RSI score (*n* = 44). **(B)** Correlation of HAMD score with RSI score (*n* = 44).

The tVNS group’s ΔRSI scores were significantly less than that for the sham-tVNS group (11.18 ± 1.26 vs. 0.23 ± 1.69, *p* < 0.001; Figure 3). Similarly, tVNS effectively improved patients’ anxiety and depression status [ΔHAMA scores (12.19 ± 1.62 vs. 0.41 ± 0.38, *p* < 0.001; ΔHAMD 10.14 ± 1.59 vs. 0.36 ± 1.29, *p* < 0.001)] compared to sham group. Furthermore, the HAMA and HAMD scores were significantly lower in the tVNS group compared to the sham-tVNS group at the end of treatment (4.86 ± 1.08 vs. 16.00 ± 1.86, *p* < 0.001; 4.82 ± 1.30 vs. 14.41 ± 2.03, *p* < 0.001; Figures 4A,B). Interestingly, the RSI score of the patients before treatment were positively correlated with HAMA (r = 0.623; *p* < 0.001) and HAMD (r = 0.593; *p* < 0.001; Figures 5A,B). The difference of RSI, anxiety and depression in the two groups were shown in Table 2.

**Table 2:** Effects of tVNS and sham-tVNS on throat symptom and mental health.

	RSI score			HAMA			HAMD		
	Before treatment	After treatment	Difference	Before treatment	After treatment	Difference	Before treatment	After treatment	Difference
tVNS (*n* = 22)	17.55 ± 1.85	6.36 ± 1.59	11.18 ± 1.26	17.05 ± 2.30	4.86 ± 1.08	12.19 ± 1.62	14.95 ± 2.36	4.82 ± 1.30	10.14 ± 1.59
sham-tVNS (*n* = 22)	17.86 ± 2.21	17.64 ± 1.36	0.23 ± 1.69	16.41 ± 2.02	16.00 ± 1.86	0.41 ± 0.38	14.77 ± 2.45	14.41 ± 2.03	0.36 ± 1.29
t	0.52	25.24	24.40	0.34	23.67	22.85	0.25	16.86	22.43
*P*	0.61	**<0.001**	**<0.001**	0.74	**<0.001**	**<0.001**	0.803	**<0.001**	**<0.001**

tVNS, transcutaneous auricular vagus nerve stimulation. ^*^P < 0.05 vs. before treatment. RSI, reflux symptom index; HAMA, 14-item Hamilton Anxiety Scale; HAMD, 17-item Hamilton Depression Scale.

### 3.3 Effects of tVNS and sham-tVNS on high-resolution esophageal manometry

The Δ UESP of the tVNS group was significantly higher than that of the sham group (29.32 ± 10.25 vs. 2.48 ± 6.56 mmHg, *p* < 0.001). Similarly, the Δ LESP of the tVNS group was significantly higher than that of the sham group (4.31 ± 4.93 vs. −0.25 ± 2.28 mmHg, *p* = 0.01). There was no change of the UESL (5.14 ± 0.81 vs. 5.05 ± 1.01, *p* = 0.744) and LESL (2.99 ± 0.42 vs. 2.97 ± 0.37, *p* = 0.879) in tVNS group compared to sham group after the two groups received different interventions. CFV (4.52 ± 1.14 vs. 3.50 ± 0.85, delta = 1.02 ± 0.88, *p* < 0.05) and DL (7.96 ± 1.09 vs. 7.02 ± 0.91, delta = 0.94 ± 0.81, *p* < 0.05) had a significant decrease, while DCI (2047.13 ± 477.61 vs. 2453.90 ± 509.11, *p* < 0.05) and IRP (4.97 ± 1.67 vs. 8.02 ± 2.04, *p* < 0.05) had a significant increase after tVNS compared to baseline. In contrast, there was no change of the CFV (4.51 ± 0.80 vs. 4.49 ± 0.75, *p* = 0.938), DL (8.10 ± 0.92 vs. 7.90 ± 0.99, *p* = 0.501), DCI (1941.50 ± 601.33 vs. 2052.62 ± 479.63, *p* = 0.502) and IRP (4.94 ± 1.84 vs. 5.02 ± 1.56, *p* = 0.875) after sham-tVNS compared to baseline (Tables 3, 4).

**Table 3:** Effects of tVNS and sham-tVNS on high-resolution esophageal manometry.

	tVNS (*n* = 22)		sham-tVNS (*n* = 22)	
	Before treatment	After treatment	Before treatment	After treatment
UESP (mmHg)	51.80 ± 9.34	81.12 ± 12.18^*^	53.40 ± 8.84	55.88 ± 8.45
UESL (cm)	5.14 ± 0.81	5.05 ± 1.01	4.99 ± 1.08	5.07 ± 0.78
LESP (mmHg)	20.78 ± 5.47	25.09 ± 5.05^*^	20.13 ± 6.14	19.88 ± 4.77
LESL (cm)	2.99 ± 0.42	2.97 ± 0.37	2.99 ± 0.43	2.95 ± 0.34
CFV (cm/s)	4.52 ± 1.14	3.50 ± 0.85^*^	4.51 ± 0.80	4.49 ± 0.75
DL (s)	7.96 ± 1.09	7.02 ± 0.91^*^	8.10 ± 0.92	7.90 ± 0.99
DCI (mmHg•cm•s)	2047.13 ± 477.61	2453.90 ± 509.11^*^	1941.50 ± 601.33	2052.62 ± 479.63
IRP (mmHg)	4.97 ± 1.67	8.02 ± 2.04^*^	4.94 ± 1.84	5.02 ± 1.56

tVNS, transcutaneous auricular vagus nerve stimulation; UESP, upper esophageal sphincter pressure; UESL, upper esophageal sphincter length; LESP, lower esophageal sphincter pressure; LESL, lower esophageal sphincter length; CFV, contraction front velocity; DL, distal latency; DCI, distal contraction integral; IRP, integrated relaxation pressure. ^*^p < 0.05 vs. before treatment.

**Table 4:** Effects of tVNS and sham-tVNS on high-resolution esophageal manometry.

	Before treatment	After treatment	Difference	Before treatment	After treatment	Difference	Before treatment	After treatment	Difference	Before treatment	After treatment	Difference
	UESP (mmHg)			UESL (cm)			LESP (mmHg)			LESL (cm)		
tVNS (*n* = 22)	51.80 ± 9.34	81.12 ± 12.18	29.32 ± 10.25	5.14 ± 0.81	5.05 ± 1.01	0.09 ± 0.48	20.78 ± 5.47	25.09 ± 5.05	4.31 ± 4.93	2.99 ± 0.42	2.97 ± 0.37	0.02 ± 0.34
sham-tVNS (*n* = 22)	53.40 ± 8.84	55.88 ± 8.45	2.48 ± 6.56	4.99 ± 1.08	5.07 ± 0.78	0.08 ± 0.46	20.13 ± 6.14	19.88 ± 4.77	−0.25 ± 2.28	2.99 ± 0.43	2.95 ± 0.34	0.04 ± 0.32
t	0.76	12.12	15.28	0.58	0.43	0.39	0.17	4.56	5.29	0.08	0.19	0.22
*P*	0.53	<0.001	<0.001	0.73	0.78	0.81	0.88	0.03	0.01	0.93	0.85	0.81
	**CFV (cm/s)**			**DL (s)**			**DCI (mmHg•cm•s)**			**IRP (mmHg)**		
tVNS (*n* = 22)	4.52 ± 1.14	3.50 ± 0.85	1.02 ± 0.88	7.96 ± 1.09	7.02 ± 0.91	0.94 ± 0.81	2047.13 ± 477.61	2453.90 ± 509.11	406.77 ± 327.21	4.97 ± 1.67	8.02 ± 2.04	3.05 ± 1.26
sham-tVNS (*n* = 22)	4.51 ± 0.80	4.49 ± 0.75	−0.02 ± 0.66	8.10 ± 0.92	7.90 ± 0.99	0.14 ± 0.62	1941.50 ± 601.33	2052.62 ± 479.63	111.12 ± 408.89	4.94 ± 1.84	5.02 ± 1.56	0.08 ± 1.19
t	0.08	4.93	5.02	0.19	3.98	4.02	0.58	11.28	15.59	0.53	9.98	10.26
*P*	0.93	**0.02**	**0.02**	0.86	**0.04**	**0.04**	0.73	**<0.001**	**<0.001**	0.77	**<0.001**	**<0.001**

tVNS, transcutaneous auricular vagus nerve stimulation; UESP, upper esophageal sphincter pressure; UESL, upper esophageal sphincter length; LESP, lower esophageal sphincter pressure; LESL, lower esophageal sphincter length; CFV, contraction front velocity; DL, distal latency; DCI, distal contraction integral; IRP, integrated relaxation pressure. ^*^P < 0.05 vs. before treatment.

### 3.4 Effects of tVNS and sham-tVNS on autonomic functions

**Figure 6:**
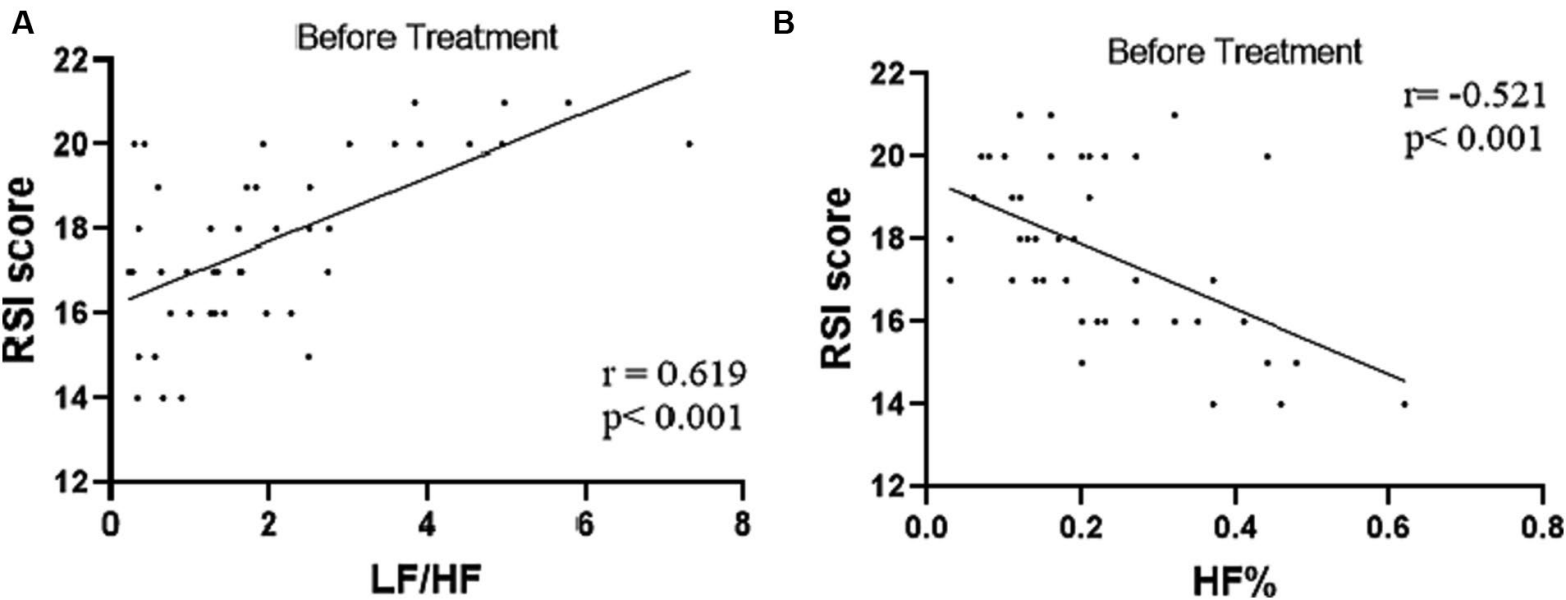
**(A)** Correlation of LF/HF with RSI score (*n* = 44). **(B)** Correlation of HF% with RSI score (*n* = 44).

**Figure 7:**
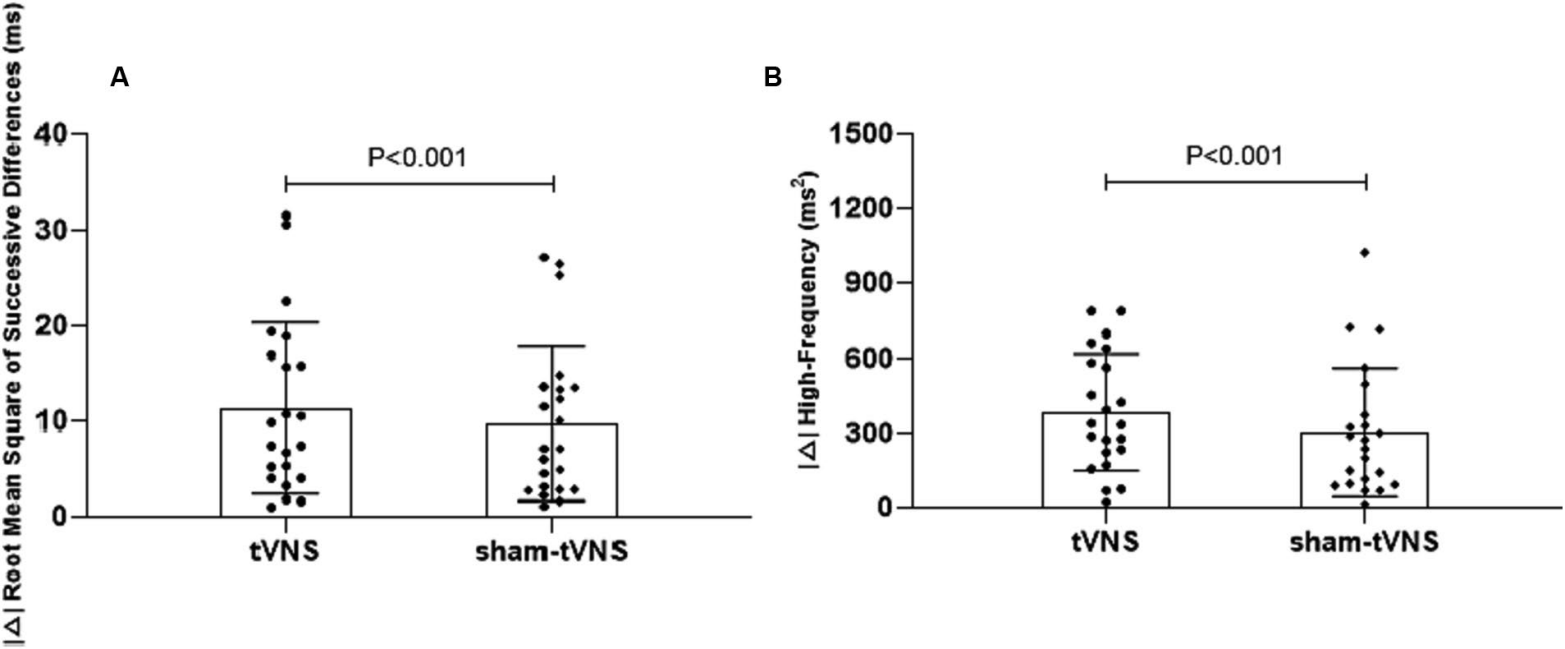
**(A)** The effect of tVNS (*n* = 22) compared with sham-tVNS (*n* = 22) on delta RMSSD. ^*^p < 0.05 vs. before treatment. **(B)** Effect of tVNS (*n* = 22) and sham-tVNS (*n* = 22) on delta HF. ^*^p < 0.05 vs. before treatment.

Combined RSI and HRV spectral analysis prior to receiving tVNS and sham-tVNS showed that RSI was positively correlated with LF/HF (r = 0.619; *p* < 0.001) and negatively correlated with HF% (r = −0.521; *p* < 0.001; Figures 6A,B). The Δ RMSSD of the tVNS group was significantly higher than that of the sham group (6.16 ± 3.37 vs. 3.56 ± 2.13 ms, *p* < 0.050). Similarly, the Δ HF of the tVNS group was significantly higher than that of the sham group (259.98 ± 40.26 vs. 8.82 ± 3.40 ms^2^, *p* < 0.05; Figures 7A,B). The difference of autonomic functions in the two groups were shown in Table 5.

**Table 5:** Effects of tVNS and sham-tVNS on autonomic functions.

	RMSSD			HF		
	Before treatment	After treatment	Difference	Before treatment	After treatment	Difference
tVNS (*n* = 22)	40.54 ± 12.30	46.70 ± 11.87	6.16 ± 3.37	596.46 ± 59.86	856.44 ± 39.40	259.98 ± 40.26
sham-tVNS (*n* = 22)	36.86 ± 11.85	40.42 ± 12.23	3.56 ± 2.13	598.44 ± 59.63	607.26 ± 57.25	8.82 ± 3.40
t	2.17	12.48	10.28	0.18	23.56	20.18
*P*	0.38	**<0.001**	**<0.001**	0.59	**<0.001**	**<0.001**

tVNS, transcutaneous auricular vagus nerve stimulation. ^*^P < 0.05 vs. before treatment. RMSSD, root mean square of successive differences; HF, high frequency.

## 4 Discussion

Some methods employing vagus nerve stimulation (VNS) has garnered approval as an intervention for various conditions, including epilepsy, depression, and migraine (Ryvlin et al., 2014; Beh and Friedman, 2019; Bottomley et al., 2020; Farmer et al., 2021). Recently, the impact of VNS on patients with functional dyspepsia was investigated, employing the SNM-FDC01 device to transcutaneously stimulate the vagus nerve at the bilateral auricular cymba concha regions. Through a comprehensive assessment encompassing acute and chronic trials, tVNS exhibited the capacity to enhance vagal nerve activity, thereby ameliorating gastric accommodation and motility. These effects resulted in marked improvements in the significant dyspeptic symptoms experienced by patients (Zhu et al., 2021). Farmer et al. (2021) proposed a set of minimal reporting items to guide future tVNS studies. The suggested items address specific technical aspects of the device and stimulation parameters. It is necessary to study and define standardized protocols for treatment, since still studies are often using inhomogeneous study designs and stimulation parameters.

The current study underscores the efficacy of noninvasive tVNS treatment targeting the auricular branch of the vagus nerve. This was administered externally through electrodes, conducted twice daily over a 2-week duration, and yielded noteworthy enhancements in both pharyngeal discomfort and mental well-being among patients grappling with LPRD. Notably, this endeavor was undertaken as a randomized, single-blind, sham-controlled pilot trial, ensuring the subjects remained uninformed about the specific treatment allocation. Moreover, objective measurements, encompassing UES pressure and autonomic functions, revealed substantial improvements in individuals who received active tVNS in comparison to those subjected to sham-tVNS. Previous study demonstrated that plasma melatonin concentration could be increased by tVNS, thus relieving peripheral neuropathic pain (Wang et al., 2022). Meanwhile, multiple tVNS sessions are antidiabetic in diabetes through triggering of tidal secretion of melatonin (Li et al., 2014; Wang et al., 2015).

While acid suppression therapy stands as the favored therapeutic approach for LPRD, its efficacy may be compromised in certain patients due to intricate factors including dietary and sleep patterns (Cui et al., 2019; Lechien et al., 2021). In this context, the current investigation highlights that significant amelioration of LPRD symptoms, as ascertained by the RSI score, transpired over a span of 2 weeks following tVNS intervention. Notably, Belafsky et al. suggest RSI scores surpassing 13 indicate the likelihood of LPRD, and the subsequent reduction below this threshold post-tVNS suggests resolution of the condition (Morice et al., 2022). Insight from prior studies also suggests a close interplay between depression, anxiety, and the occurrence of LPRD (Liu et al., 2022). Indeed, Caparroz et al. (2019) propose that LPRD patients often grapple with anxiety and/or depression attributed to compromised autonomic regulatory function. In congruence with these observations, the present study unveils a positive correlation between reflux symptoms and both anxiety and depressive symptoms, manifesting as significant associations between RSI scores and scores from the HAMA or the HAMD. This empirical discovery aligns with research by Huang et al. (2022). Remarkably, the application of tVNS led to a pronounced reduction in anxiety and depression scores among LPRD patients. These effects are likely attributed to the central impacts of tVNS, which have been noted in previous studies involving patients with neurological disorders (Redgrave et al., 2018; Sun et al., 2022).

The UES and LES constitute vital elements of the esophageal-pharyngeal mechanical barrier. The LES, functioning as the primary defense against reflux, inhibits the retrograde flow of gastroduodenal contents into the esophagus. Simultaneously, the UES safeguards the upper pharyngeal region against the ingress of these contents. In the context of diminished LES function, a compensatory elevation in UES resting pressure can mitigate potential reflux incidents, an anatomical foundation paramount in LPRD (Hunt et al., 1970). Factors such as age-related changes and esophageal pathologies may precipitate UES relaxation, facilitating reflux into the pharyngeal area, thereby perpetuating a cycle whereby symptomatology impacts UES contractile function, creating a self-perpetuating loop (Vardouniotis et al., 2009). Subsequent to short-term or sustained exposure to esophageal acid, diastolic reflex in the esophagus intensifies, while contractile reflex weakens, contributing to UES relaxation (Lang et al., 2019). Altered vagal modulation can disrupt LES and UES function, a phenomenon evident in cases of vagal hypoplasia (Wang et al., 2019). Studies by Szczesniak et al. (2011) and Ranjbar et al. (2022) revealed substantial pressure irregularities in the LES and UES of LPRD patients, underlining the prevalence of comorbid esophageal dyskinesia in these individuals (Szczesniak et al. 2011; Ranjbar et al. 2022). Our previous study reported that transcutaneous electrical acustimulation improved the reflux symptoms in GERD patients by increasing LESP, which may be mediated via the autonomic and enteric mechanisms (Yu et al., 2019).

In a similar vein, HRV spectral analysis proved instrumental in quantifying parasympathetic and sympathetic activity (Pachon-M et al., 2020). This approach, applied to LPRD patients, unveiled a positive correlation between sympathetic activity and the Reflux Symptom Index (RSI), coupled with a negative correlation between parasympathetic activity and RSI. Such findings align with prior research by Wang et al. (2019). While acknowledging this, it’s important to underscore that our study centered exclusively on LPRD patients, precluding direct comparison with healthy volunteers, thus preventing definitive confirmation of autonomic nervous function impairment in LPRD patients. Reduced parasympathetic activity and sympathetic imbalance are implicated in conditions like refractory gastroesophageal reflux disease (rGERD) and functional outlet obstructive constipation (FOOC; Liu et al., 2020). Strategies aimed at enhancing parasympathetic activity might offer efficacy in addressing these disorders (Liu et al., 2020; Yang et al., 2022). Meanwhile, our latest basic study reported that tVNS significantly improved the constipation-predominant irritable bowel syndrome symptoms (Liu et al., 2024). Interestingly, the current study demonstrated tVNS-induced elevation of parasympathetic activity among LPRD patients compared to baseline. Prior research utilizing functional magnetic resonance imaging (fMRI) analysis highlighted increased blood-oxygen-level-dependent (BOLD) signals in brain regions such as the postcentral gyrus, bilateral insula, frontal cortex, operculum, and cerebellum in response to tVNS. These responses may be attributed to the stimulation of afferent fibers of the auricular branch of the vagus nerve and in turn a possible modulation of efferent parasympathetic fibers via the brain. This mechanistic cascade culminates in amplified proximal esophageal constriction (Badran et al., 2018; Tsou et al., 2021).

## 5 Limitations

Notwithstanding, this pilot clinical inquiry entails several limitations. It functions as a single-blind study conducted within a single center, involving a relatively small sample size. The absence of long-term follow-up observations necessitates cautious interpretation of the study’s results, as they may not entirely encapsulate the prolonged effects of tVNS treatment in the entirety of LPRD patients. As a means to rectify these limitations, the prospect of executing a large-scale, multicenter, double-blind pilot study emerges to yield a more comprehensive comprehension of the response of LPRD patients to tVNS treatment. Notably, the cavity concha was found to not only be innervate by the vagus nerve in earlier studies (Peuker and Filler, 2002), which may have a certain impact on the results. Finally, we acknowledge that false positives were not controlled for via statistical corrections, though the study’s exploratory nature entailed numerous, related statistical comparisons.

## 6 Conclusion

In summation, the present short-term tVNS intervention yielded improvements in pharyngeal discomfort and mental well-being, concomitant with heightened UES pressure and augmented parasympathetic activity. These enhancements are likely underpinned by intricate autonomic and esophageal mechanisms. Offering a needleless, self-administered approach, tVNS emerges as a potentially accessible and cost-effective adjunctive therapy for individuals with LPRD. However, further exploration is requisite to uncover the cellular and molecular pathways through which tVNS mitigates LPRD and its enduring effects on patients.

## Data availability statement

The original contributions presented in the study are included in the article/supplementary material, further inquiries can be directed to the corresponding author.

## Ethics statement

The studies involving humans were approved by Ethics Committee of the First Affiliated Hospital of USTC (Registration No: 2016-L36). The studies were conducted in accordance with the local legislation and institutional requirements. The participants provided their written informed consent to participate in this study.

## Author contributions

YH: Writing – original draft, Writing – review & editing. JL: Writing – original draft, Writing – review & editing. CL: Writing – original draft, Writing – review & editing. CS: Writing – review & editing. MM: Writing – review & editing. SL: Writing – review & editing. YY: Writing – original draft, Writing – review & editing.
